# Prescribing tamoxifen in primary care for the prevention of breast cancer: a national online survey of GPs’ attitudes

**DOI:** 10.3399/bjgp17X689377

**Published:** 2017-02-14

**Authors:** Samuel G Smith, Robbie Foy, Jennifer A McGowan, Lindsay C Kobayashi, Andrea DeCensi, Karen Brown, Lucy Side, Jack Cuzick

**Affiliations:** Institute of Health Sciences, University of Leeds, Leeds, and Wolfson Institute of Preventive Medicine, Queen Mary University of London, London.; Institute of Health Sciences, University of Leeds, Leeds.; Institute of Epidemiology and Healthcare; Institute for Women’s Health, University College London, London.; Center for Population and Development Studies, Harvard TH Chan School of Public Health, Harvard University, Cambridge, MA.; Division of Medical Oncology, Ospedali Galliera, Genoa, and Wolfson Institute of Preventive Medicine, Queen Mary University of London, London.; Department of Cancer Studies, University of Leicester, Leicester.; Institute for Women’s Health, University College London, London.; Wolfson Institute of Preventive Medicine, Queen Mary University of London, London.

**Keywords:** breast cancer, chemoprevention, general practice, preventive therapy, primary care, tamoxifen

## Abstract

**Background:**

The cancer strategy for England (2015–2020) recommends GPs prescribe tamoxifen for breast cancer primary prevention among women at increased risk.

**Aim:**

To investigate GPs’ attitudes towards prescribing tamoxifen.

**Design and setting:**

In an online survey, GPs in England, Northern Ireland, and Wales (*n* = 928) were randomised using a 2 × 2 between-subjects design to read one of four vignettes describing a healthy patient seeking a tamoxifen prescription.

**Method:**

In the vignette, the hypothetical patient’s breast cancer risk (moderate versus high) and the clinician initiating the prescription (GP prescriber versus secondary care clinician [SCC] prescriber) were manipulated in a 1:1:1:1 ratio. Outcomes were willingness to prescribe, comfort discussing harms and benefits, comfort managing the patient, factors affecting the prescribing decision, and awareness of tamoxifen and the National Institute for Health and Care Excellence (NICE) guideline CG164.

**Results:**

Half (51.7%) of the GPs knew tamoxifen can reduce breast cancer risk, and one-quarter (24.1%) were aware of NICE guideline CG164. Responders asked to initiate prescribing (GP prescriber) were less willing to prescribe tamoxifen than those continuing a prescription initiated in secondary care (SCC prescriber) (68.9% versus 84.6%, *P*<0.001). The GP prescribers reported less comfort discussing tamoxifen (53.4% versus 62.5%, *P* = 0.01). GPs willing to prescribe were more likely to be aware of the NICE guideline (*P* = 0.039) and to have acknowledged the benefits of tamoxifen (*P*<0.001), and were less likely to have considered its off-licence status (*P*<0.001).

**Conclusion:**

Initiating tamoxifen prescriptions for preventive therapy in secondary care before asking GPs to continue the patient’s care may overcome some prescribing barriers.

## INTRODUCTION

In the UK more than 53 000 women are diagnosed with breast cancer each year, and 11 000 die of the disease.^1^ Women with a family history of the disease are at increased risk, and this accounts for 5–10% of all breast cancer cases.^2^ The majority of women with an increased risk of breast cancer are ineligible for prophylactic surgery, and therefore prevention by other means is a priority.^3^

In 2013, the UK National Institute for Health and Care Excellence (NICE) issued recommendations regarding the use of two selective oestrogen-receptor modulators (SERMs), tamoxifen and raloxifene, for women at increased risk of breast cancer because of their family history.^3^ SERMs reduce breast cancer incidence by ≥30%.^4^ The number needed to treat to prevent one diagnosis of breast cancer in the first 10 years is 42. However, the decision to prescribe SERMs is complicated because current preventive therapy trials are not designed to detect effects on mortality,^5^ and the medications are not licensed for primary prevention. SERMs also increase the risk of thromboembolic events, endometrial cancer, and menopausal side effects.^4^ Only one in six women accept the offer of breast cancer preventive therapy, and uptake is significantly lower in non-trial settings.^6^ The cancer strategy for England (2015–2020) has recommended that action be taken to ensure preventive therapy is appropriately prescribed in the NHS.^7^

Previous qualitative work by the authors has suggested GPs and family history clinicians experience barriers to implementing the NICE clinical guideline for familial breast cancer (CG164).^8^ Concerns were raised relating to licensing, interpretation of the NICE guideline, and responsibility for prescribing. GPs suggested they may be more comfortable continuing a preventive therapy prescription, providing it had been initiated in secondary care. To validate and quantify these findings, the authors surveyed a national sample of GPs who were randomised to view one of four case studies of a hypothetical patient seeking a tamoxifen prescription for primary prevention.

## METHOD

### Study design and sample

A national survey of GPs practising in the UK was undertaken in April 2016. Members of a research panel with more than 33 000 members were e-mailed an invitation to take part. Sampling was done by inviting panellists on an unfiltered random basis to avoid over-sampling. GPs practising in Scotland were excluded from these analyses because an agreed care pathway already exists there for the prescription of tamoxifen.^9^ GPs practising outside of the UK were excluded. The study was prospectively registered (ISRCTN14292000).

**How this fits in**

The cancer strategy for England recommends that GPs prescribe tamoxifen for breast cancer primary prevention among women at increased risk. The authors demonstrated that GPs are largely unaware of using tamoxifen for primary prevention, and a significant minority may be unwilling to prescribe the drug for eligible patients. These data show that a shared care agreement between primary and secondary care could alleviate a number of concerns, and facilitate appropriate prescribing.

### Questionnaire design

Responders were randomised in a 1:1:1:1 ratio to one of four case study vignettes describing a hypothetical patient at increased risk of breast cancer (Appendix 1). The vignettes were designed with input from clinical geneticists, medical oncologists, GPs, and public health specialists. They were intended to be representative of a typical patient attending a family history clinic, and were informed by the authors’ earlier research.^8^ The vignettes described a hypothetical patient’s age (45 years), risk level, premenopausal status, her lack of contraindications, and her discussion in secondary care. The case studies were presented using a between-subjects 2 × 2 factorial design, where patient risk level (moderate lifetime risk of 17–30% versus high lifetime risk of ≥30%) and the clinician responsible for initiating the prescription (GP versus secondary care clinician) were manipulated. The secondary care clinician was described as a family history clinician. The case study was available to them throughout the survey.

Prior to the vignettes, responders were informed about the NICE guidelines, the eligibility criteria for tamoxifen, the harms and benefits of the drug, the typical patient pathway, and the licensing status. This information was available throughout the survey.

### Measures

#### Chemoprevention awareness

Responders were asked if they were aware tamoxifen could be used for risk reduction in women with a family history of breast cancer, and if they were aware of the relevant NICE guideline. Responders answering ‘yes’ to the second question were asked how they became aware that tamoxifen could be used for primary prevention. Example options are shown in Appendix 2.

#### Willingness to prescribe

GPs’ willingness to prescribe tamoxifen was assessed, and response options were ‘definitely not willing’, ‘probably not willing’, ‘probably willing’, and ‘definitely willing’. Data were combined to reflect unwilling and willing responses.

#### Comfort discussing harms and benefits of long-term management

GPs were asked to report their comfort in discussing the harms and benefits of tamoxifen with a patient, as well as their comfort in managing the patient for the duration of the prescription. Response options were ‘very uncomfortable’, ‘quite uncomfortable’, ‘quite comfortable’, and ‘very comfortable’. Data were combined to reflect GPs who were uncomfortable and comfortable.

#### Barriers to prescribing

Responders were offered a series of factors that could potentially affect the willingness of GPs to write a prescription for the hypothetical patient. Responders were provided with the response categories ‘strongly disagree’, ‘disagree’, ‘agree’, and ‘strongly agree’. Data were combined to reflect agreement and disagreement.

#### Responder characteristics

GPs self-reported their sex, age in 10-year bands, status within the practice, region of practice, year qualified in general practice, and special interests.

### Statistical analysis

The data were described using percentages. For the vignettes, the main effects of risk and type of prescriber on willingness to prescribe, comfort discussing tamoxifen, and comfort managing the patient were tested using unadjusted logistic regression. Logistic regression models with the interaction between risk and prescriber were also tested. Multivariable logistic regression adjusted for nation, GP status, sex, age, experience, and specialisms was used to compare subgroup differences on study outcomes. Unadjusted logistic regression was used to compare differences in endorsement of barriers between GPs who were and were not willing to prescribe tamoxifen. Statistical significance was set at *P*<0.05. Analyses were conducted using SPSS (version 22).

## RESULTS

### Sample overview

In total, 13 764 of approximately 33 000 GPs were approached via e-mail, and 1321 started the survey (9.6%). Responders were excluded if they did not agree to the terms and conditions (*n* = 35), did not complete the survey (*n* = 143), completed the survey after the deadline (*n* = 35), or failed a data quality check (*n* = 101). Scottish GPs (*n* = 79) were also excluded, leaving data from 928 GPs for this analysis. An overview of the sample compared with national data is shown in Table 1. Participant characteristics across the study arms were comparable (Table 2).

**Table 1.** GP sample (*n* = 928) compared with national data

	**Sample, %**	**National data,^a^ %**
**Country**		
England	92.9	82.8
Wales	4.2	4.7
Northern Ireland	2.9	2.7

**Occupation**		
GP partner	57.8	67.6
Salaried/locum GP	39.2	21.2
GP retainers	0.2	0.9
GP specialist trainee	2.0	10.3
Other	0.8	–

**Sex**		
Male	57.3	50.8
Female	42.7	49.2

**Age, years**		
<50	72.3	57.2
≥50	27.7	38.0

**Experience, years**		
0–10	44.1	–
>10	55.9	–

**Specialisms**		
Cancer	12.7	–
Preventive medicine	14.3	–
Family history	5.2	–
Genetics	3.2	–

a Data from British Medical Association briefing document.^10^ If Scotland were included in the sample, the proportions in each country are as follows: England 85.6%, Scotland 7.8%, Wales 3.9%, and Northern Ireland 2.7%.

**Table 2.** GP sample (*n* = 928) across the study arms

	**High risk, GP first prescriber (*n*= 175), *n* (%)**	**Moderate risk, GP first prescriber (*n*= 252), *n* (%)**	**High risk, GP second prescriber (*n*= 251), *n* (%)**	**Moderate risk, GP second prescriber (*n*= 250), *n* (%)**
**Country**				
England	163 (93.1)	231 (91.7)	231 (92.0)	237 (94.8)
Wales	7 (4.0)	13 (5.1)	10 (4.0)	9 (3.6)
Northern Ireland	5 (2.9)	8 (3.2)	10 (4.0)	4 (1.6)

**Occupation**				
GP partner	104 (59.4)	142 (56.3)	141 (56.2)	149 (59.6)
Salaried/locum GP	69 (39.4)	99 (39.3)	104 (41.4)	92 (36.8)
GP retainers	0 (0)	0 (0)	1 (0.4)	1 (0.4)
GP specialist trainee	1 (0.6)	8 (3.2)	4 (1.6)	6 (2.4)
Other	1 (0.6)	3 (1.2)	1 (0.4)	2 (0.8)

**Sex**				
Male	100 (57.1)	146 (57.9)	140 (55.8)	146 (58.4)
Female	75 (42.9)	106 (42.1)	111 (44.2)	104 (41.6)

**Age, years**				
<50	126 (72.0)	184 (73.0)	185 (73.7)	176 (70.4)
≥50	49 (28.0)	68 (27.0)	66 (26.3)	74 (29.6)

**Experience, years**				
0–10	70 (40.0)	117 (46.4)	105 (41.8)	117 (46.8)
>10	105 (60.0)	135 (53.6)	146 (58.2)	133 (53.2)

**Specialisms^a^**				
Cancer	26 (14.9)	37 (14.7)	26 (10.4)	29 (11.6)
Preventive medicine	34 (19.4)	33 (13.1)	26 (10.4)	40 (16.0)
Family history	12 (6.9)	11 (4.4)	7 (2.8)	18 (7.2)
Genetics	9 (5.1)	9 (3.6)	3 (1.2)	9 (3.6)

a Specialism responses indicate proportions indicating they had a special interest in that field. Therefore figures do not compute to 100%. Note: all GPs from Scotland were randomised to the ‘high risk, GP prescriber’ condition, as per the national guideline in that country. They were not included in these analyses.

### Awareness of tamoxifen and the NICE guidelines

**Figure 1.**
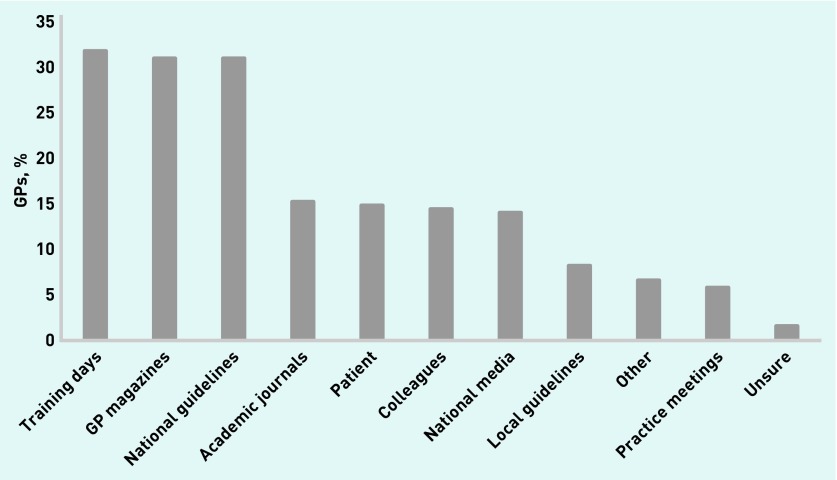
***GPs’ sources of information about tamoxifen (n = 243).***

Approximately half (51.7%) of the responders were aware tamoxifen could be used to reduce the risk of breast cancer, and one-quarter (24.1%) were aware of NICE guideline CG164. Among those who were aware of the NICE guideline, common sources of information about tamoxifen were training days (31.7%), GP magazines (30.9%), and the NICE guideline (30.9%) (Figure 1).

### Barriers to prescribing and discussing breast cancer preventive therapy

#### Willingness to prescribe

The majority of GPs (77.4%) were willing to prescribe tamoxifen for the hypothetical patient (definitely willing 17.6%, probably willing 59.8%). The remaining GPs were either probably not willing (18.1%) or not at all willing (4.5%) to prescribe tamoxifen. Male GPs were more likely to report a willingness to prescribe tamoxifen than female GPs (odds ratio [OR] 1.38, 95% confidence interval [CI] = 1.00 to 1.90, *P* = 0.05). Willingness to prescribe was unaffected by the other GP characteristics (Table 3).

**Table 3.** GPs’ willingness to prescribe tamoxifen by responder characteristics (*n* = 928)

**Characteristic**	**Willing, %**	**OR (95% CI)**	***P*-value**
**Country**			
England	77.0	Ref	Ref
Wales	76.9	1.06 (0.49 to 2.29)	0.89
Northern Ireland	88.9	2.45 (0.72 to 8.34)	0.15

**GP status (*n*= 919)**			
GP partner	78.0	0.95 (0.68 to 1.33)	0.77
Salaried/locum GP	76.4	Ref	Ref
GP specialist trainee	73.7	0.91 (0.32 to 2.64)	0.86

**Sex**			
Male	79.9	1.38 (1.00 to 1.90)	0.05
Female	74.0	Ref	Ref

**Age, years**			
<50	75.4	Ref	Ref
≥50	82.5	1.41 (0.92 to 2.14)	0.11

**Experience, years**			
0–10	74.6	Ref	Ref
>10	79.6	1.18 (0.82 to 1.70)	0.38

**Cancer specialism**			
Yes	81.4	1.36 (0.78 to 2.34)	0.28
No	76.8	Ref	Ref

**Preventive medicine specialism**			
Yes	81.2	1.24 (0.73 to 2.10)	0.43
No	76.7	Ref	Ref

**Family history specialism**			
Yes	75.0	0.49 (0.22 to 1.11)	0.09
No	77.5	Ref	Ref

**Genetics specialism**			
Yes	83.3	1.75 (0.59 to 5.20)	0.32
No	77.2	Ref	Ref

OR = odds ratio.

Table 4 shows the proportion of GPs willing to prescribe tamoxifen in each condition. GPs told they would be asked to be the first prescriber were significantly less willing to prescribe tamoxifen than GPs told they would be asked to continue a prescription initiated in secondary care (68.9% versus 84.6%, OR 0.40, 95% CI = 0.29 to 0.55, *P* <0.001). There were no differences in responders’ willingness according to patient risk (moderate risk 77.1% versus high risk 77.7%, OR 1.04, 95% CI 0.76 to 1.41, *P* = 0.83). There was no interaction between the two factors.

**Table 4.** Willingness to prescribe tamoxifen within each condition

	**High risk, % (*n*= 426)**	**Moderate risk, % (*n*= 502)**	**GP prescriber, % (*n*= 427)**	**Secondary care prescriber, % (*n*= 501)**
Definitely willing	16.7	18.3	13.6	21.0
Probably willing	61.0	58.8	55.3	63.7
Probably not willing	18.5	17.7	24.4	12.8
Not at all willing	3.8	5.2	6.8	2.6

#### Comfort in discussing harms and benefits of tamoxifen

The majority of GPs were either very comfortable (6.5%) or quite comfortable (51.8%) discussing the harms and benefits of tamoxifen. The remaining GPs were either quite uncomfortable (36.6%) or very uncomfortable (5.1%). In multivariable analysis, comfort in discussing the harms and benefits of tamoxifen with a patient was higher among GPs >50 years (OR 1.53, 95% CI = 1.08 to 2.17, *P* = 0.02), with >10 years’ experience (OR 1.39, 95% = CI 1.02 to 1.91, *P* = 0.04), and those with a special interest in cancer (OR 1.79, 95% CI = 1.12 to 2.85, *P* = 0.02). Comfort discussing tamoxifen was unaffected by the remaining GP characteristics (Appendix 3).

GPs were more likely to report they were comfortable in discussing the harms and benefits of tamoxifen if they were told a secondary care clinician would write the first prescription, compared with those who were told they would be asked to prescribe first (62.5% versus 53.4%, OR 0.69, 95% CI = 0.53 to 0.90, *P* = 0.01). There were no significant differences in reported comfort discussing the harms and benefits according to the patient’s risk (moderate risk 56.6% versus high risk 60.3%, *P* = 0.25), and there was no interaction between the two factors.

#### Comfort in managing the patient’s care

The majority of GPs were very comfortable (7.8%) or quite comfortable (58.6%) managing the patient, should she decide to take tamoxifen. The remaining GPs were quite uncomfortable (29.8%) or very uncomfortable (3.8%). Comfort managing the hypothetical patient was higher among GPs with a special interest in preventive medicine (OR 1.66, 95% CI = 1.03 to 2.69, *P* = 0.04). Comfort managing the patient was unaffected by all other GP characteristics (Appendix 4).

There were no differences in comfort managing the patient comparing the prescriber manipulation or the patient risk manipulation. There was also no interaction between these variables.

#### Tamoxifen attitudes according to knowledge of the national guideline

GPs who were aware of the NICE guideline were more willing to prescribe tamoxifen, with 82.4% who were aware being willing to prescribe, compared with 75.7% who were unaware (OR 1.50, 95% CI = 1.02 to 2.19, *P* = 0.04). Awareness of the NICE guideline also affected reported comfort in discussing the potential harms and benefits of tamoxifen, with 66.5% of those who were aware being comfortable, compared with 55.6% of those who were unaware being comfortable (OR 1.58, 95% CI = 1.16 to 2.17, *P*<0.01). There was no difference in comfort in managing the patient according to awareness of the guidelines (OR 1.25, 95% CI = 0.90 to 1.73, *P* = 0.18).

#### Factors affecting prescribing decisions

GPs were most likely to agree that the evidence for the benefits of the drug (95.0%), the existence of the NICE guideline (95.0%), and the patient’s awareness of the harms and benefits (94.1%) affected their decision (Table 5). GPs who were willing to prescribe were more likely to consider a number of factors than those who were unwilling. Key differences were observed with regard to their consideration of prescribing off label (91.4% of those unwilling to prescribe agreed that it affected their decision, compared with 69.6% of those willing to prescribe, OR 4.65, 95% CI = 2.8 to 7.73, *P*<0.001), the patient’s awareness of the harms and benefits (unwilling 81.9%, willing 97.6%, OR 9.11, 95% CI = 5.02 to 16.53, *P*<0.001) and the evidence for the benefits of the drug (unwilling 87.6%, willing 97.2%, OR 4.93, 95% CI = 2.69 to 9.03, *P*<0.001).

**Table 5.** Factors affecting the decision to prescribe tamoxifen for patient (% agreement, *n* = 928)

	**Willingness to prescribe**			
	**Overall, %**	**Unwilling, %**	**Willing, %**	***P*-value**
Evidence for the benefits of the drug	95.0	87.6	97.2	<0.001
The existence of NICE guideline (or national equivalent)	95.0	87.6	97.2	<0.001
Patient awareness of possible harms and benefits	94.1	81.9	97.6	<0.001
The patient’s level of risk for breast cancer	93.8	82.9	96.9	<0.001
Patient interest in taking tamoxifen	90.6	74.8	95.3	<0.001
GPs’ confidence in their knowledge of tamoxifen	89.5	83.3	91.4	0.001
Evidence for the harms of the drug	89.3	89.0	89.4	0.880
The patient’s support from the family history clinician^a^	88.6	69.0	94.3	<0.001
First prescription being made by family history clinician	86.0	72.7	88.4	<0.001
Policy of GPs’ clinical commissioning group	80.2	82.4	79.5	0.360
Prescribing off-label	74.6	91.4	69.6	<0.001
The first prescription being made by GP	71.9	85.0	66.0	<0.001
Attitudes of colleagues at the same career stage	61.6	57.6	32.8	0.170
Attitudes of more senior colleagues	59.4	58.1	59.7	0.670
Prescribing budget in GPs’ general practice	42.1	41.4	42.3	0.810
Financial costs of tamoxifen	41.4	37.6	42.5	0.210

a This item was only asked of those GPs allocated to the relevant condition. NICE = National Institute for Health and Care Excellence.

## DISCUSSION

### Summary

The cancer strategy for England (2015–2020) has recommended that work should be done to ensure tamoxifen is appropriately prescribed as preventive therapy to interested patients. This national study showed that only three-quarters of UK GPs reported that they would be willing to prescribe tamoxifen for a hypothetical patient at increased risk of breast cancer. Willingness was significantly lower among GPs who were told that they would be asked to initiate the drug prescription, compared with GPs who were asked to continue a prescription from a clinician in secondary care. Levels of reported comfort in discussing the harms and benefits of tamoxifen were low, and responders who were asked to prescribe first reported significantly lower levels of comfort. The most commonly reported barrier among GPs who were unwilling to prescribe was concern about off-label prescribing.

### Strengths and limitations

This study was strengthened by its randomised design and large national sample. The authors were able to compare the sample with the UK GP workforce,^10^ which showed that the current sample were more likely to be salaried GPs, younger, and male. Recruitment was from an online panel, and not all UK GPs are affiliated with the company responsible. The response rate was low, which may further limit generalisability. Multiple barriers to prescribing tamoxifen were investigated, and therefore the possibility of a type I error is increased. The patient vignette was designed to be representative of a typical patient in this context, but specific characteristics may not match all patients. Similarly, the healthcare professional was described as a family history clinician, and attitudes towards prescribing may have been different if alternative clinical positions were described. The vignette was hypothetical, and prescribing behaviour may be different in a clinical setting.

### Comparison with existing literature

The authors’ previous qualitative work suggested that a shared care agreement between primary and secondary care would reduce ambiguity for prescribing, and encourage discussions about preventive therapy with high-risk patients.^8^ The current study’s data support this conclusion.

Their earlier work also suggested GPs are concerned about the lack of licence for tamoxifen when used for prevention.^8^ The survey responses showed that this is considered in the decision making of GPs, but other factors had a greater influence. Together, the interview and survey data help to explain why uptake of preventive therapy is lower in routine clinical settings compared with trial participation.^6^

### Implications for practice

Guidance for prescribing tamoxifen in Scotland has been produced,^9^ but there is no formal care pathway for the rest of the UK. Developing a pathway involving both primary and secondary care in a shared care agreement could substantially increase GPs’ willingness to prescribe. Although GPs may become more familiar with tamoxifen as a preventive agent over time, shared care agreements could form one facet of a longer-term implementation strategy. Consideration would, however, have to be given to the fact that genetic counsellors do not have prescribing rights, and therefore a supervising clinician would have to be responsible for prescribing in secondary care. The approach described is similar to the national prescribing policy developed within the Health Improvement Scotland guidance for tamoxifen.^11^ The authors recommend that NHS England, NHS Wales, and the Department of Health in Northern Ireland should replicate and adapt the Scottish guidelines.

One of the major barriers to implementing the tamoxifen guidelines is the low awareness of its potential to be used as preventive therapy. Although cross-sectional surveys do not allow causal inferences, the data suggest increasing awareness of preventive medications could facilitate appropriate prescribing behaviour. The most common sources of information were training days, GP magazines, and national guidelines. Strategies to promote awareness of tamoxifen for primary prevention should consider ways to target these sources. Providing an up-to-date and accurate source of information for GPs so they are prepared to have informed conversations with patients may reduce prescribing barriers. Although local decision aids are currently in use, a single national resource could ensure all patients are provided with the same information.

Developing standardised pro-formas for secondary care clinicians to send to GPs when referring patients to discuss preventive therapy could be a useful strategy to improve GP awareness. These pro-formas could be adapted from those included in the Health Improvement Scotland guidelines.^9^ These data suggest that encouraging GPs to consider the evidence for the benefits of the drug may encourage prescribing. Perceiving that patients may be lacking awareness of the harms and benefits of tamoxifen was also shown to be a barrier to prescribing among GPs. Highlighting that harms and benefits have already been communicated to the patient by a specialist may alleviate these concerns.

These data suggest the lack of licence for tamoxifen is a factor in decision making, and is the most commonly reported barrier among those who are unwilling to prescribe. One strategy to overcome anxieties related to off-label prescribing is through acknowledgement in the *British National Formulary* (*BNF*). Although the *BNF* does not have the authority to license a medication, it frequently describes alternative unlicensed indications for medications. The authors suggest that primary prevention is listed as an indication for tamoxifen in the *BNF* for the appropriate patient groups.

## Appendix 1. Case studies describing hypothetical patient at increased risk of breast cancer

**Table:**

	**GP writes first prescription**	**Secondary care clinician writes first prescription**
Moderate:17–30% lifetime risk	Sarah is a 45-year-old woman with a family history of breast cancer. She consulted you previously and was referred to a local family history clinic for risk assessment. A family history clinician assessed her as having a moderate risk of breast cancer. This means she has a lifetime risk of between 17% and 30%. Sarah has discussed the potential harms and benefits of taking tamoxifen for 5 years with the family history clinician. She has expressed an interest in taking tamoxifen. Sarah is premenopausal with no menstrual dysfunction, is not planning pregnancy, has no contraindications, and is taking no other medications. The family history clinician supports her decision to take tamoxifen and has also referred her for additional screening. The family history clinician requested that you write the first prescription and continue to act as the main prescriber.	Sarah is a 45-year-old woman with a family history of breast cancer. She consulted you previously and was referred to a local family history clinic for risk assessment. A family history clinician assessed her as having a moderate risk of breast cancer. This means she has a lifetime risk of between 17% and 30%. Sarah has discussed the potential harms and benefits of taking tamoxifen for 5 years with the family history clinician. She has expressed an interest in taking tamoxifen. Sarah is premenopausal with no menstrual dysfunction, is not planning pregnancy, has no contraindications, and is taking no other medications. The family history clinician supports her decision to take tamoxifen and has also referred her for additional screening. The family history clinician has written the first prescription, and has requested that you take over as the main prescriber.
High: 30% lifetime risk	Sarah is a 45-year-old woman with a family history of breast cancer. She consulted you previously and was referred to a local family history clinic for risk assessment. A family history clinician assessed her as having a high risk of breast cancer. This means she has a lifetime risk of ≥30%. Sarah has discussed the potential harms and benefits of taking tamoxifen for 5 years with the family history clinician. She has expressed an interest in taking tamoxifen. Sarah is premenopausal with no menstrual dysfunction, is not planning pregnancy, has no contraindications, and is taking no other medications. The family history clinician supports her decision to take tamoxifen and has also referred her for additional screening. The family history clinician requested that you write the first prescription and continue to act as the main prescriber.	Sarah is a 45-year-old woman with a family history of breast cancer. She consulted you previously and was referred to a local family history clinic for risk assessment. A family history clinician assessed her as having a high risk of breast cancer. This means she has a lifetime risk of ≥30%. Sarah has discussed the potential harms and benefits of taking tamoxifen for 5 years with the family history clinician. She has expressed an interest in taking tamoxifen. Sarah is premenopausal with no menstrual dysfunction, is not planning pregnancy, has no contraindications, and is taking no other medications. The family history clinician supports her decision to take tamoxifen and has also referred her for additional screening. The family history clinician has written the first prescription, and has requested that you take over as the main prescriber.

## Appendix 2. Online national survey sent to GPs in April 2016

Before we start, we are interested in some basic information about you and your practice to ensure the study is relevant for you.

S1 Please confirm your specialty.
  - GP
  - Other
S1_2 Please confirm the region you practise in.
  - England
  - Scotland
  - Wales
  - Northern Ireland
S10 Do you have a role in commissioning?
  - Yes
  - No

### Chemoprevention

We are interested in the use of chemoprevention drugs in the NHS. In this instance, chemoprevention is the use of medication to lower the risk of cancer in people *not previously* affected by the disease. We are particularly interested in chemoprevention using tamoxifen and aspirin.

We have supplied some information below for you to read, with some follow-up questions. Please take your time to read this information.

### Tamoxifen: the essentials

NICE guidelines suggest women at high risk of breast cancer (≥30% lifetime risk) should be offered tamoxifen for primary prevention and clinicians should consider offering the drug to moderate-risk women (17–30% lifetime risk). Women would take tamoxifen for 5 years. Tamoxifen can reduce the risk of getting breast cancer among women at increased risk by at least one third. Women taking tamoxifen have an increased risk of endometrial cancer, venous thromboembolic events, and menopausal side effects. There is no generally accepted nationwide care pathway for prescribing tamoxifen for primary prevention. Tamoxifen will usually be discussed with patients in secondary care, and interested patients will typically be referred back to primary care. Tamoxifen is not licensed for a primary prevention indication, and therefore prescriptions are made off-label.

### Tamoxifen: the facts

#### Background

In 2013, NICE endorsed the use of tamoxifen as a therapeutic agent for women at increased risk of breast cancer because of a family history of the disease (see Clinical Guideline 164: *Familial breast cancer: classification, care and managing breast cancer and related risks in people with a family history of breast cancer*). The guidelines suggested that women at high risk of breast cancer (≥30% lifetime risk) should be offered tamoxifen, and that clinicians should consider offering tamoxifen to women at moderate risk of breast cancer (17–30% lifetime risk). Women initiating therapy would take the medication for up to 5 years.

#### Benefits and harms

A meta-analysis of women at increased risk of breast cancer who were taking selective oestrogen-receptor modulators (SERMs) (the class of medication that includes tamoxifen) showed a relative risk reduction of 38% for breast cancer incidence. Women taking tamoxifen had a higher rate of endometrial cancer (hazard ratio [HR] = 2.18), venous thromboembolic events (HR = 1.60), and cataracts (HR = 1.10). Tamoxifen can also increase the likelihood of experiencing gynaecological, sexual, and vasomotor symptoms, as well as weight gain and headaches.

#### Prescribing

There is no generally accepted nationwide care pathway for prescribing tamoxifen for primary prevention. Patients at increased risk of breast cancer will generally be seen in clinical genetics clinics, family history clinics, or breast cancer departments. The option of tamoxifen will be discussed in secondary care, and patients who are interested in taking tamoxifen will typically be referred back to primary care. Tamoxifen is not licensed for a primary prevention indication, and therefore all prescriptions have to be made off-label.

Please confirm you have read and understood the above by clicking on the next button.

Q1 Before today, were you aware that tamoxifen can be used to reduce the risk of breast cancer in women with a family history of the disease?
  - Yes
  - No
Q1a Before today, were you aware of the NICE clinical guidelines or Health Improvement Scotland guidelines outlining recommendations regarding the use of tamoxifen for primary prevention?
  - Yes
  - No
Q1b How did you first become aware that tamoxifen could be used to reduce the risk of breast cancer in women with a family history of the disease?
  **Table:**
  Tick all that apply	Yes	No
  Previously raised by a patient		
  Training days/educational meetings		
  Academic journals		
  GP magazines, for example, *Pulse*		
  Informal discussion with colleagues		
  National media		
  Local guidelines		
  National guidelines (for example, NICE or national equivalent)		
  Practice meetings		
  Other (please specify)		
  Unsure		

See Appendix 1 for description of the profiles.

Q2 Would you be willing to write the prescription for Sarah?
  - Not at all willing
  - Probably not willing
  - Probably willing
  - Definitely willing
Q2a Would you want to speak with anyone else before you decided whether to write this prescription?
  - Yes
  - No
Q2b Please describe who you would want to speak with, and why, before you decided whether to write this prescription.
Q4 A number of factors have been identified in interviews with GPs that could influence whether they would be willing to prescribe tamoxifen. How much do you **agree** or **disagree** that the following factors affected your decision of whether or not to write a prescription for Sarah?
  **Table:**
  	**Strongly disagree**	**Disagree**	**Agree**	**Strongly agree**
  The evidence for the benefits of the drug				
  The evidence for the harms of the drug				
  Prescribing off-label because tamoxifen is not licensed for primary prevention				
  The first prescription being made by a family history clinician				
  The first prescription being made by you				
  The financial costs of tamoxifen				
  Sarah’s level of risk for breast cancer				
  Sarah’s interest in taking tamoxifen				
  Sarah’s awareness of the possible harms and benefits				
  Your confidence in your knowledge of tamoxifen				
  Sarah’s support from the family history clinician				
  The attitudes of your colleagues who are at the same career stage as you				
  The attitudes of your colleagues who are more senior than you				
  The prescribing budget in your general practice				
  The policy of your clinical commissioning group				
  The existence of NICE guidelines (or national equivalent)				
  Are there any other factors not listed here that you believe would influence your decision making? (Please specify) If you do not have any further comments, please type ‘n/a’				
Q3 How comfortable would you feel discussing the possible benefits and harms of tamoxifen with Sarah?
  - Very uncomfortable
  - Quite uncomfortable
  - Quite comfortable
  - Very comfortable
Q3a If Sarah started taking tamoxifen, how comfortable would you feel managing her care for the duration of the prescription?
  - Very uncomfortable
  - Quite uncomfortable
  - Quite comfortable
  - Very comfortable
Q4a Do you have any comments regarding the prescription of tamoxifen for women at increased risk of breast cancer?
  If you do not have any further comments, please type ‘n/a’
Q5 Has your clinical commissioning group discussed the use of tamoxifen for primary prevention?
  - Yes
  - No
  - Don’t know
Q5a What was the outcome?
  - Local policy in place
  - Still under discussion
  - No policy formed
  - Unsure
  - Other (please describe)
Q6 In your opinion, who is responsible for making local policy decisions regarding the use of tamoxifen for primary prevention?
  - Local clinical commissioning groups
  - Your own general practice
  - Local medicines management group, drug and therapeutic committee, or equivalent
  - Other (please specify)
Q6b In your opinion, would clinical commissioning groups have any concerns about GPs prescribing tamoxifen for primary prevention?
  - Yes
  - No
Q6c What concerns do you think the clinical commissioning groups would have about GPs prescribing tamoxifen for primary prevention?

## Appendix 3. Comfort discussing the harms and benefits of tamoxifen by responder characteristics (*n* = 928)

**Table:**

**Characteristic**	**Comfortable, %**	**OR (95% CI)**	***P*-value**
**Country**			
England	58.4	Ref	Ref
Wales	51.3	0.75 (0.39 to 1.45)	0.40
Northern Ireland	66.7	1.40 (0.61 to 3.21)	0.43

**GP status (*n*= 919)**			
GP partner	60.1	1.07 (0.80 to 1.46)	0.67
Salaried/locum GP	55.2	Ref	Ref
GP specialist trainee	57.9	1.30 (0.50 to 3.36)	0.59

**Sex**			
Male	60.0	1.10 (0.83 to 1.46)	0.49
Female	56.1	Ref	Ref

**Age, years**			
<50	54.2	Ref	Ref
≥50	68.9	1.53 (1.08 to 2.17)	0.02

**Experience, years**			
0–10	51.3	Ref	Ref
>10	63.8	1.39 (1.02 to 1.91)	0.04

**Cancer specialism**			
Yes	70.3	1.79 (1.12 to 2.85)	0.02
No	56.5	Ref	Ref

**Preventive medicine specialism**			
Yes	67.7	1.44 (0.92 to 2.25)	0.11
No	56.7	Ref	Ref

**Family history specialism**			
Yes	62.5	0.63 (0.30 to 1.31)	0.22
No	58.1	Ref	Ref

**Genetics specialism**			
Yes	63.3	1.09 (0.46 to 2.59)	0.85
No	58.1	Ref	Ref

## Appendix 4. Comfort managing the patient by responder characteristics (*n* = 928)

**Table:**

**Characteristic**	**Comfortable, %**	**OR (95% CI)**	***P*-value**
**Country**			
England	65.9	Ref	Ref
Wales	74.4	1.51 (0.72 to 3.17)	0.28
Northern Ireland	70.4	1.35 (0.58 to 3.18)	0.49

**GP status (*n*= 919)**			
GP partner	67.7	1.08 (0.80 to 1.46)	0.61
Salaried/locum GP	64.8	Ref	Ref
GP specialist trainee	47.4	0.51 (0.20 to 1.31)	0.16

**Sex**			
Male	67.3	1.05 (0.79 to 1.40)	0.75
Female	65.2	Ref	Ref

**Age, years**			
<50	64.1	Ref	Ref
≥50	72.4	1.35 (0.94 to 1.94)	0.11

**Experience, years**			
0–10	63.8	Ref	Ref
>10	68.4	1.06 (0.77 to 1.47)	0.72

**Cancer specialism**			
Yes	72.9	1.30 (0.81 to 2.10)	0.28
No	65.4	Ref	Ref

**Preventive medicine specialism**			
Yes	75.9	1.66 (1.03 to 2.69)	0.04
No	64.8	Ref	Ref

**Family history specialism**			
Yes	66.7	0.48 (0.23 to 1.01)	0.05
No	66.4	Ref	Ref

**Genetics specialism**			
Yes	80.0	2.13 (0.77 to 5.83)	0.14
No	65.9	Ref	Ref
